# The Association of Apathy With Incident Dementia: A Multiple Mediation Analysis of Cardiovascular Risk Factors

**DOI:** 10.1002/gps.70092

**Published:** 2025-05-10

**Authors:** Josephine E. Lindhout, Jan Willem van Dalen, Willem A. van Gool, Edo Richard, Marieke P. Hoevenaar‐Blom

**Affiliations:** ^1^ Department of Public and Occupational Health Amsterdam UMC Amsterdam the Netherlands; ^2^ Department of General Practice Amsterdam UMC Amsterdam the Netherlands; ^3^ Department of Neurology Amsterdam UMC Amsterdam the Netherlands; ^4^ Department of Neurology Donders Centre for Brain Behaviour and Cognition Radboud University Medical Center Nijmegen the Netherlands

**Keywords:** apathy, dementia, multiple mediation analysis, prevention, risk factor

## Abstract

**Objectives:**

Despite established links between apathy, cardiovascular disease, and dementia, it remains unclear if cardiovascular risk factors (CVRF) play a mediating role in the association between apathy and dementia. If apathy increases dementia risk via lifestyle‐related dementia risk factors, targeted lifestyle interventions could help high‐risk individuals.

**Methods:**

We used data from the preDIVA study including 3303 individuals aged 70–78 years. Apathy was assessed using the geriatric depression scale, and CVRF (cardiovascular risk factors) (systolic blood pressure, cholesterol, diabetes, body mass index (BMI), smoking, and physical activity) were considered as potential mediators. Outcome was incident dementia during 12 years of follow‐up. We assessed mediation using Multiple Mediation Analysis (MMA).

**Results:**

Of the association between apathy and dementia (HR 1.49 [95% CI 0.99–2.41]), 27% was mediated by physical inactivity, BMI and diabetes combined. Of this total, physical inactivity mediated 28% of the effect (HR 1.12, 95% CI 1.03–1.29), diabetes 9% of the effect (HR 1.04, 95% CI 1.02–1.10), and BMI counteracted these effects by −12% (HR 0.95, 95% CI 0.88–0.98).

**Conclusion:**

The relationship between apathy and dementia is partly mediated by physical inactivity, BMI and diabetes. Apathy is an important clinical marker that signals the existence of potentially modifiable pathways, providing an opportunity for lifestyle interventions. To potentially reduce dementia risk via lifestyle modification in patients with apathy, a tailored approach should be taken to overcome the characterizing symptom of diminished motivation.

**Summary**

- The link between apathy and dementia is partially explained by physical inactivity, BMI, and diabetes.
- Apathy may serve as a crucial clinical sign, indicating the presence of modifiable pathways and opportunities for lifestyle interventions.
- To reduce dementia risk in patients with apathy, a personalized approach is needed to address their diminished motivation and encourage lifestyle changes.

## 1 Introduction

Apathy is a syndrome of disturbed motivation, manifesting in diminished interest, cognition, and emotional expression [1]. Symptoms of apathy can occur in the context of depression, but also independently as a separate syndrome. Approximately 25%–44% of cognitively healthy community‐dwelling older individuals are thought to have symptoms of apathy [2, 3, 4, 5]. Apathy has a direct and substantial negative impact on both patients and their social environment [6, 7]. Cognitively healthy community‐dwelling adults with symptoms of apathy are at increased risk of developing dementia as compared to those without [8, 9]. The mechanisms underlying this association are unknown. Since apathy is a common symptom of dementia, apathy in older people may partly be a prodromal sign of developing dementia [10], and therefore not necessarily causally related to dementia risk. Another part of the relation between apathy and dementia may be explained by apathy being associated with individuals' unhealthy lifestyles, which in turn increases dementia risk [11]. Physical inactivity, hypertension, obesity, dyslipidemia, diabetes, and smoking are all independently associated with an increased risk of dementia [12]. Apathy may induce these conditions and their risk factors via symptoms of reduced motivation [13], decreased engagement in health behaviors [11] or treatment adherence [14, 15]. Distinguishing between these prodromal and potentially modifiable pathways in the relationship between apathy and dementia is important, because this may offer leads for prevention: if the association between apathy and an increased dementia risk is due to unhealthy lifestyle, then individuals with apathy may specifically benefit from lifestyle interventions. This is especially important because individuals with apathy may tend to retract from self‐care, and therefore form a group that might particularly benefit from active prevention strategies. Furthermore, clues about the extent to which apathy may be prodromal rather than causally related to incident dementia may help elucidating the mechanisms potentially underlying apathy in old age, which may be relevant for developing apathy treatments. We hypothesize that the increased risk of incident dementia in individuals with symptoms of apathy may be due to the association of apathy with cardiovascular risk factors. Therefore, we aim to investigate to which extent the relationship between apathy and increased risk of dementia is due to lifestyle‐related dementia risk factors.

## 2 Materials and Methods

### 2.1 Design, Population and Setting

This study used data from the preDIVA study, a randomized controlled trial including community‐dwelling older adults (*n* = 3526) between 70 and 78 years of age at baseline [16]. The initial study was approved by the medical ethics committee of the Academic Center, Amsterdam, Netherlands. Participants gave written informed consent. This trial studied the effect of a 6–8 years multi‐domain intervention on incident dementia and the study was extended with an observational follow‐up period up to 12 years. In this study all community dwelling adults registered with one of 116 participating general practitioners were invited. Exclusion criteria were presence of possible dementia, terminal illness or any other condition likely to hinder successful 6‐year follow‐up according to the general practitioner (e.g., cancer or alcoholism). After completion of the trial no significant effect of the intervention on dementia incidence was found after both the initial trial and the observational extension period [16, 17]. In the current context data are analyzed as a prospective cohort study. More information on details of the preDIVA study can be found elsewhere [16].

### 2.2 Exposure, Mediators and Covariates

Exposure, mediators and covariates were all measured at baseline. We used the 3‐item apathy subscale of the 15‐item Geriatric Depression Scale [18] (GDS) to assess apathy, as done in previous studies [2, 3, 4, 5, 8]. This sub scale was based on previous factor analyses, and includes the items: “Have you dropped many of your activities and interest? (yes/no: 1/0 point)”, “Do you prefer to stay at home, rather than going out and doing new things? (yes/no: 1/0 point)” and “Do you feel full of energy? (yes/no: 0/1 point)” [2]. Apathy was operationalized as scoring on ≥ 2 of these items. The mediating factors included in the analyses were systolic blood pressure (SBP) in mmHg, total cholesterol (TC) in mmol/L, and body mass index (BMI) in kg/m^2^ as continuous variables, as well as physician‐diagnosed diabetes mellitus (DM), self‐reported current smoking, and physical activity (PA), which was classified as sufficient to meet the WHO guidelines for physical activity, as dichotomous variables. Assessment of risk factors was done by a trained nurse at baseline visit at the general practice [16]. Anthropometrics and blood pressure were obtained using a standardized protocol and blood samples were collected for lipid spectrum and blood glucose analysis. Self‐reported presence of risk factors (smoking, medication use and cardiovascular history) was cross checked with patients' electronic health records (EHR). Covariates were age, sex, level of education and history of cardiovascular disease (CVD).

### 2.3 Outcome

Cognitive status and functioning in daily life were assessed in person every 2 years using the Mini‐Mental State Examination (MMSE) [19] and the Academic Medical Center Linear Disability Score (ALDS) [20], respectively. Indications for the presence of dementia was based on these measurements, complemented by information from the general practitioner, hospital admissions and outpatient diagnostic evaluations from EHR, and the National Death Registry during the trial phase (first 6–8 years) [16]. During the observational extension phase, dementia was established using a step‐wise approach. First, individuals were contacted by telephone, asked whether they had a dementia diagnosis, and administered the telephone interview of cognitive status (TICS) [21]. The conventional TICS cut‐off to indicate possible dementia is < 27 points. In individuals who had a TICS score > 30 and who indicated that they did not have dementia or cognitive problems, we recorded “no dementia”. For individuals with a TICS score ≤ 30, or those who indicated that they did have dementia or cognitive impairment, we accessed the EHR from their general practitioner to assess whether they had been diagnosed with dementia [16, 17, 22]. All dementia diagnoses were ascertained and re‐evaluated after 1 year by a blinded outcome adjudication committee consisting of clinicians.

### 2.4 Statistical Analyses

First, we tested for differences between individuals with and without symptoms of apathy, using independent samples student's *T*‐test, Chi‐square test or the Mann‐Whitney‐U test as appropriate [23]. Second, we explored the association of apathy with the individual risk factors using logistic or linear regression as appropriate and the association of the individual mediators with incident dementia using Cox regression. We performed multiple mediation analysis (MMA) using the MMA package in *R* [24]. This method uses simulation with bootstrapping to estimate the mediation effects, and can be applied to binary, categorical and continuous types of exposure, mediator and outcome variables. To identify mediators, the pathway from exposure to mediator and from mediator to outcome were tested. The association of apathy with individual risk factors is tested with ANOVA for continuous, and with chi‐square tests for dichotomous variables. The association of a mediator with incident dementia is tested with Type III tests in a Cox model including all potential mediators and covariates [25]. A Type III test checks for evidence of significant difference in survival functions across strata for dichotomous variables and for a unit increase for continuous variables. The results then indicate if variables qualify as mediators and provide effect sizes of the separate indirect effects, which should be interpreted as the hazard ratio (HR) that is explained by the mediator. Confidence intervals of the effect sizes are based on bias corrected bootstrap methods [26]. Our main MMA model was adjusted for age, sex, level of education and history of CVD. The second model was further adjusted for treatment arm in the RCT, antihypertensive medication and cholesterol lowering medication. For sensitivity analyses we have repeated the multiple mediation analysis excluding individuals with a total GDS‐15 score > 6, as this may be indicative of depression. Secondly, we excluded individuals diagnosed with dementia during the first year, to exclude cases in which apathy was an early symptom of dementia. All statistical analyses were performed in Rstudio version 4.2.1.

## 3 Results

A total of 3303 (94%) participants had complete data on the relevant mediators and covariates, and were included in the mediation analysis. During the median follow‐up time of 10.3 years (interquartile range (IQR) 6.9–10.9), 391 (12%) participants were diagnosed with dementia and 974 (29%) died, yielding an incidence rate of 0.013 new cases per person years. Participants with symptoms of apathy (14%) were more likely to be female, currently smoking and having a history of CVD and diabetes, and were less educated and less active compared to those without. Also participants with apathy had a higher GDS‐15 score at baseline (Table 1).

**TABLE 1:** Baseline characteristics of the total population and stratified by apathy status.

Characteristic	All	No apathy	Apathy	*p*‐value
*N*	3303	2835	468	
Female, *N*(%)	1800 (54.5)	1512 (53.3)	288 (61.5)	0.001
Age (years), mean (SD)	74.34 (2.50)	74.28 (2.50)	74.74 (2.44)	< 0.001
History of CVD^a^, *N*(%)	977 (29.6)	796 (28.1)	181 (38.7)	< 0.001
MMSE^b^ score, (median [IQR])	28.00 [27.00, 29.00]	28.00 [27.00, 29.00]	28.00 [27.00, 29.00]	0.018
GDS‐15^c^ score, (median [IQR])	1.00 [0.00, 2.00]	1.00 [0.00, 2.00]	4.00 [3.00, 7.00]	< 0.001
Education^d^, *N*(%)				< 0.001
Basic	800 (24.2)	650 (22.9)	150 (32.0)	
Intermediate	1867 (56.6)	1622 (57.2)	245 (52.4)	
Advanced	636 (19.3)	563 (19.9)	73 (15.6)	
Systolic blood pressure (mmHg), mean(SD)	155.22 (21.35)	155.37 (21.32)	154.30 (21.52)	0.313
Cholesterol (mmol/L), mean(SD)	5.23 (1.10)	5.24 (1.09)	5.17 (1.12)	0.209
Body Mass index (kg/m^2^), mean(SD)	27.44 (4.15)	27.30 (3.99)	28.27 (4.93)	< 0.001
Diabetes, *N*(%)	591 (17.9)	478 (16.9)	113 (24.1)	< 0.001
Current smoking, *N*(%)	435 (13.2)	335 (11.8)	100 (21.4)	< 0.001
Physically inactive^e^, *N*(%)	610 (18.5)	439 (15.5)	171 (36.5)	< 0.001

*Note:* We tested for differences between those with and without symptoms of apathy.

^a^ History of cardiovascular disease excluding stroke or transient ischemic attack.

^b^ Mini Mental State Examination.

^c^ Geriatric Depression Scale, for *n* = 5 data was missing for MMSE and GDS‐15 score.

^d^ Basic is primary education. Intermediate is upper secondary or post‐secondary non‐tertiary education. Advanced is pre‐university, higher professional education or university.

^e^ Physically active is operationalized as being adherent to international World Health Organization guidelines for physical activity.

Explorative analysis of the association of apathy with potential mediators, adjusted for age, sex, level of education and history of CVD showed that apathy was significantly associated with physical inactivity (OR 2.90, 95% CI 2.33–3.60), higher BMI (mean difference 0.80, 95%CI 0.40–1.21 kg/m^2^), diabetes (OR 1.46, 95% CI 1.15–1.84) and current smoking (OR 1.97, 95% CI 1.52–2.53), but not with total cholesterol (mean difference −0.03, 95% CI −0.13–0.07 mmol/L) nor systolic blood pressure (mean difference −0.77, 95% CI −2.87–1.33 mmHg) (Table 2). Analysis of the association of potential mediators with incident dementia showed that only physical inactivity was significantly associated with a higher hazard of developing dementia (HR 1.38, 95%CI 1.09–1.76) (Table 3).

**TABLE 2:** Association of apathy with individual mediators.

Continuous risk factor as outcome	Mean difference for apathy (95% CI)
Systolic blood pressure (mmHg)	−0.77 (−2.87–1.33)
Total cholesterol (mmol/L)	−0.03 (−0.13–0.07)
Body Mass index (kg/m^2^)	0.80 (0.40–1.21)^a^
Binary risk factor as outcome	OR of apathy (95% CI)
Physically inactivity	2.90 (2.33–3.60)^a^
Diabetes mellitus	1.46 (1.15–1.84)^a^
Current smoking	1.97 (1.52–2.53)^a^

*Note:* All models were adjusted for age, sex, level of education, history of CVD. For models with a continuous risk factor as outcome this means that patients with apathy on average have a higher BMI (+0.80 kg/m^2^, 95 CI 0.40–1.21).

^a^ Significant based on confidence intervals.

**TABLE 3:** Association of individual mediators with incident dementia.

Mediator	Hazard ratio dementia (95% CI)
Systolic blood pressure (mmHg)	0.99 (0.99–1.00)
Total cholesterol (mmol/L)	1.02 (0.92–1.12)
Body Mass index (kg/m^2^)	0.98 (0.95–1.00)^a^
Physical inactivity	1.38 (1.09–1.76)^a^
Diabetes mellitus	1.27 (0.99–1.63)
Current smoking	0.86 (0.61–1.22)

*Note:* All Cox models were adjusted for age, sex, level of education, history of CVD.

^a^ Significant based on confidence intervals.

### 3.1 MMA

**FIGURE 1:**
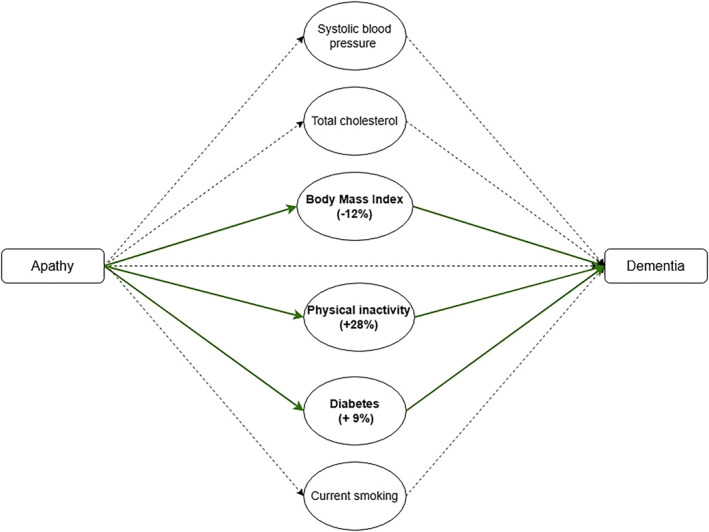
Multiple mediation analysis of cardiovascular risk factors in the association between apathy and dementia. Solid lines indicates significant pathways, dashed lines insignificant pathways.

In the MMA, overall, individuals with apathy had a 49% higher hazard of incident dementia (total effect HR: 1.49, 95% CI 0.99–2.41). Combined, the mediated effects explained 27% of the association between apathy and incident dementia (indirect HR 1.11, 95% CI 1.01–1.27 compared to the total HR of 1.49). Three variables were identified as significant mediators for the relationship between apathy and incident dementia: physical inactivity (proportion mediation (PM): 28%), BMI (PM: −12%) and diabetes (PM: 9%) (Table 4, Figure 1). The remaining “direct effect” of apathy on incident dementia (i.e. not explained by the included mediators) was a 34% increased dementia hazard (direct HR 1.34, 95% CI 0.92–2.17), making up 73% of the total association between apathy and incident dementia. Results of the fully adjusted MMA model adjusting for intervention status, AHM and cholesterol lowering medication were comparable to main results (Table 4). Sensitivity analysis excluding patients with a GDS‐15 > 6 showed comparable results (Supporting Information S1). Sensitivity analysis excluding individuals diagnosed with dementia during the first year showed comparable results (Supporting Information S1).

**TABLE 4:** Multiple mediation analysis showing total, direct and indirect effects of physical activity, diabetes and BMI in the association of apathy on dementia incidence.

Effect	MMA model 1		MMA model 2	
	HR (95% CI)	Proportion of total effect^a^	HR (95% CI)	Proportion of total effect^a^
Total	1.49 (0.99–2.41)		1.49 (0.99–2.50)	
Direct	1.34 (0.92–2.17)	73%	1.35 (0.91–2.22)	75%
Total indirect	1.11 (1.01–1.27)^b^	27%	1.10 (0.99–1.26)	25%
Body Mass index (kg/m^2^)	0.95 (0.88–0.98)^b^	−12%	0.95 (0.87–0.98)^b^	−13%
Diabetes	1.04 (1.02–1.10)^b^	9%	1.03 (1.00–1.09)^b^	7%
Physical inactivity	1.12 (1.03–1.29)^b^	28%	1.12 (1.02–1.29)^b^	28%

*Note:* Model is bootstrapped 1000 times. Indirect effects are mediated via physical activity, diabetes and BMI. Model 1 is adjusted for age, sex, level of education, history of cardiovascular disease. Model 2 is additionally adjusted for intervention status and use of antihypertensive and cholesterol lowering medication use at baseline. Due to correlation in mediator effects, direct and indirect effect do not add up to the total indirect effect [24].

^a^ Proportion mediation is regression coefficient of the direct or indirect effect divided by regression coefficient of the total effect.

^b^ Significant based on confidence intervals.

## 4 Discussion

This study aimed to investigate to what extent the relationship between apathy and increased risk of dementia is mediated by lifestyle‐related dementia risk factors. In total 27% of the association between apathy and incident dementia was explained by diabetes, physical inactivity, and BMI combined. The higher prevalence of diabetes and physical inactivity in patients with symptoms of apathy explained respectively 9% and 28% of the total association with dementia. Of the total association between apathy and dementia, a higher mean BMI in those with symptoms of apathy mitigated 12% of the total effect.

In older adults, apathy, independent of depression, is a known risk factor for incident CVD [27]. Apathy could render people less inclined to lead a healthy lifestyle, since it is characterized by reduced motivation and goal directed behavior. For instance, it is known that apathy is associated with diminished physical activity and a higher behavioral risk factor score consisting of multiple risk factors (physical inactivity, smoking, alcohol use, unhealthy diet) [11]. Our study corroborates these findings by showing that patients with apathy have a higher odds of being physically inactive, and with a proportion mediation of 28%, physical activity was the strongest mediator of the relationship between apathy and dementia. Adherence to treatment for other cardiovascular risk factors may be suboptimal in people with apathy symptoms. Glycemic control is worse in patients with apathy [14]. In our study, participants with apathy had a higher odds of having diabetes and the association of apathy with dementia was partially mediated by the presence of diabetes. We found apathy to be associated to a higher mean BMI, potentially the consequence of more physical inactivity in these individuals, and this association alleviated the effects of apathy on dementia with 12%. This finding is in line with previous studies, showing that a lower or declining BMI in late life is a risk factor for dementia [28, 29]. Beforehand we hypothesized that mechanisms of reduced motivation and self–care behaviors in apathetic patients could lead to suboptimal control of blood pressure and cholesterol. However, in our study we did not find a significant relationship of cholesterol nor systolic blood pressure with either apathy or dementia. The literature on the relationship between apathy and blood pressure is heterogeneous. Associations with both higher and lower blood pressure have been reported [30, 31]. In the current study we found that apathy was associated with almost a double odds of current smoking, but smoking did not significantly increase the hazard of dementia. In a previous analysis of this cohort smoking mediated the relationship between apathy and risk of incident cardiovascular disease with 4.5% [32]. However, studies specifically addressing the relationship between smoking and apathy are scarce.

Symptoms of apathy may occur in the context of other psychiatric conditions like depression, Parkinson's disease schizophrenia and dementia. However, apathy also occurs as a standalone syndrome in cognitively healthy older people that is strongly associated with elevated dementia risk, even in individuals without depressive symptoms [8, 9]. A previous analysis of this cohort showed that individuals with symptoms of apathy, but no depressive symptoms are at an increased risk of developing dementia (HR 1.26, 95% CI 1.06–1.49) [9]. Also, when repeating our analysis excluding individuals with depressive symptoms the analysis showed comparable results. This aligns with previous literature, suggesting that apathy is a separate construct and can occur independently of depression [33].

Our study had several strengths. Our study population had a relatively long total follow‐up of up to 12 years (median 10.3 years). Medical history was cross‐checked with participants' electronic health records, minimizing the risk of recall or reporting bias. Dementia ascertainment was 99% complete and all cases were evaluated by a blinded outcome adjudication committee, therefore we performed a complete case analysis [16, 17]. Even though we cannot exclude residual confounding, the fact that the adjusted model hardly changed the (total, direct and indirect) effects indicates the robustness of our findings. There are some limitations to consider. For one, although our study population was free of dementia at baseline, in some participants neurodegenerative processes may have already commenced. Our research focused on the extent to which clinically measured symptoms of apathy in older adults are related to the risk of incident dementia through lifestyle‐related risk factors. This approach aims to determine whether this group could be a suitable target for lifestyle interventions. It is plausible that a significant portion of the association between apathy and incident dementia risk, which is not explained by cardiovascular risk factors, may be attributed to apathy being a prodromal sign of (subclinical) neurodegeneration [10, 34]. Secondly, in our study we used the GDS‐3A, a three‐item subset of GDS‐15, to identify symptoms of apathy. Validation of this construct, using the more elaborate Apathy Scale [35] as reference, showed that it is very specific (88%–93%), but has a low sensitivity (29%–33%) in community‐dwelling older adults [33]. This could lead to underestimation of the number of people having symptoms of apathy. In our study population, 14% exhibited symptoms of apathy, which is slightly lower than the 25%–44% reported in other studies of cognitively healthy older adults [5, 8, 36]. One potential reason could be that individuals with prodromal dementia may lack insight into their disease and behavioral changes. Also, people may be inclined to provide socially desirable answers indicating a healthy, active lifestyle. Since the apathy questions of the GDS are self‐reported, these factors could have led to an underestimation of the true prevalence of apathy symptoms, resulting in underdiagnosis or misclassification of apathy. This misclassification might have caused an underestimation of the true relationship between apathy symptoms and incident dementia. However, our findings that the relationship between apathy and incident dementia is in part significantly explained by cardiovascular risk factors remains. Finally, due to the cross‐sectional assessment of apathy symptoms and cardiovascular risk factors this study does not provide further indications of a causal relationship between apathy and the subsequent development of cardiovascular risk factors. This is not needed to address the question to which extent the relationship between apathy and dementia is explained by the presence of cardiovascular risk factors. Our findings suggest that older adults with symptoms of apathy may benefit from targeted interventions addressing cardiovascular risk factors to help reduce the risk of dementia. While our results were derived from the general population, it is worth considering whether similar interventions could also benefit individuals experiencing apathy in the context of specific co‐morbidities, such as Parkinson's disease. For instance, a recent systematic review and meta‐analysis indicates that in Parkinson's disease, stimulating motor activity—such as through various forms of dance—can have positive effects on both quality of life and cognitive functioning [37]. However, given that apathy is characterized by reduced activity and withdrawal from care, addressing these risk factors in this group may be challenging and could require developing a highly intensive approach.

## 5 Conclusion

In older community‐dwelling persons the relationship between apathy and incident dementia is partly explained by physical inactivity, BMI and diabetes. In our study indirect effects played a significant role, which suggests that apathy increases the hazard of dementia, at least in part via its association with these risk factors. Apathy may prove an important clinical marker of individuals at increased risk of dementia due to the presence of potentially modifiable risk factors. Patients with apathy may especially benefit from more intensive prevention and care interventions since this is a vulnerable, less self‐reliant population at high risk of dementia with a larger prevention potential.

## Conflicts of Interest

The authors declare no conflicts of interest.

## Supporting information

Supporting Information S1

## Data Availability

Individual de‐identified participant data from the preDIVA trial and the observational extension are available to academic researchers, following approval of a methodologically sound research proposal by the preDIVA data sharing committee and signature of a data access agreement. Inquiries or proposals should be directed to Dr. Marieke Hoevenaar‐Blom: m.p.hoevenaarblom@amsterdamumc.nl.
