# Supplementary material for: The Association of Apathy With Incident Dementia: A Multiple Mediation Analysis of Cardiovascular Risk Factors
Source: Int J Geriatr Psychiatry. 2025 May 10;40(5):e70092. doi: 10.1002/gps.70092 (PMC12065525; doi:10.1002/gps.70092)
Supplement: Supplementary file 1 — Supporting Information S1 [file GPS-40-e70092-s001.docx]

# Supplementary material

|  | | MMA model 1 | | | MMA model 1 excluding GDS>6 | |
| --- | --- | --- | --- | --- | --- | --- |
| Effect | **HR (95% CI)** | | **Proportion of total effect*** | **HR (95% CI)** | | **Proportion of total effect*** |
| Total | 1.49 (0.99 – 2.41) | |  | 1.36 (0.98 – 1.96) | |  |
| Direct | 1.34 (0.92 – 2.17) | | 73% | 1.28 (0.93 – 1.85) | | 80% |
| Total indirect | 1.11 (1.01 – 1.27) | | 27% | 1.06 (0.99 – 1.14) | | 20% |
| Body Mass Index (kg/m2) | 0.95 (0.88 – 0.98) | | -12% | 0.96 (0.91 – 0.99) | | 10% |
| Diabetes | 1.04 (1.02 – 1.10) | | 9% | 1.02 (1.00 – 1.07) | | 6% |
| Physical inactivity | 1.12 (1.03 – 1.29) | | 28% | 1.07 (1.03 – 1.14) | | 22% |

Supplementary Table 1: Sensitivity analysis excluding the 139 persons with a GDS > 6. For reference main MMA model 1 on the left side of the table.

|  | | MMA model 1 | | | MMA model 1 excluding first year dementia diagnoses (8 cases) | |
| --- | --- | --- | --- | --- | --- | --- |
| Effect | **HR (95% CI)** | | **Proportion of total effect*** | **HR (95% CI)** | | **Proportion of total effect*** |
| Total | 1.49 (0.99 – 2.41) | |  | 1.48 (0.96 -2.33) | |  |
| Direct | 1.34 (0.92 – 2.17) | | 73% | 1.32 (0.90 – 2.05) | | 70% |
| Total indirect | 1.11 (1.01 – 1.27) | | 27% | 1.12 (1.01 – 1.27) | | 30% |
| Body Mass Index (kg/m2) | 0.95 (0.88 – 0.98) | | -12% | 0.96 (0.88 – 0.99) | | -11% |
| Diabetes | 1.04 (1.02 – 1.10) | | 9% | 1.04 (1.00 – 1.13) | | 9% |
| Physical inactivity | 1.12 (1.03 – 1.29) | | 28% | 1.12 (1.03 – 1.26) | | 29% |

Supplementary Table 2: Sensitivity analysis excluding first year dementia cases. For reference main MMA model 1 on the left side of the table.
